# Suicide risk in a representative sample of people receiving HIV care: Time to target most-at-risk populations (ANRS VESPA2 French national survey)

**DOI:** 10.1371/journal.pone.0171645

**Published:** 2017-02-13

**Authors:** Maria Patrizia Carrieri, Fabienne Marcellin, Lisa Fressard, Marie Préau, Luis Sagaon-Teyssier, Marie Suzan-Monti, Valérie Guagliardo, Marion Mora, Perrine Roux, Rosemary Dray-Spira, Bruno Spire, ANRS-VESPA2 Study Group

**Affiliations:** 1 Aix Marseille Univ, INSERM, IRD, SESSTIM, Sciences Economiques & Sociales de la Santé & Traitement de l’Information Médicale, Marseille, France; 2 ORS PACA, Observatoire régional de la santé Provence-Alpes-Côte d’Azur, Marseille, France; 3 GREPS, Psychology Institute, Lyon 2 University, 5 avenue Pierre Mendes-France, Bron, France; 4 INSERM, UMR_S1136, Pierre Louis Institute of Epidemiology and Public Health, Team Research in social epidemiology, Paris, France; 5 Sorbonne Universités, UPMC Univ Paris 06, UMR_S1136, Pierre Louis Institute of Epidemiology and Public Health, Team Research in social epidemiology, Paris, France; Yokohama City University, JAPAN

## Abstract

**Background:**

Suicide risk is high among people living with HIV (PLHIV). This study aimed to identify major correlates of suicide risk in a representative sample of PLHIV in France, in order to help target individuals who would benefit from suicide risk screening and psychiatric care.

**Methods:**

The ANRS VESPA2 cross-sectional survey (April 2011-January 2012) collected socio-demographic, medical and behavioral data from 3,022 PLHIV recruited in 73 French HIV hospital departments. The study sample comprised the 2,973 participants with available self-reported data on suicide risk (defined as having either thought about and planned to commit suicide during the previous 12 months or attempted suicide during the same period of time) and medical data on comorbidities. Weighted Poisson models adjusted for HCV co-infection and significant clinical variables were used to estimate the relationship between suicide risk and HIV transmission groups, experience with HIV disease and other psychosocial factors.

**Results:**

Suicide risk was reported by 6.3% of PLHIV in the study sample. After adjustment for HIV immunological status and HCV co-infection, women (IRR [95%CI]:1.93 [1.17; 3.19]) and men who have sex with men (MSM) (1.97 [1.22; 3.19]) had a higher suicide risk than the rest of the sample. Moreover, the number of discrimination-related social contexts reported (1.39 [1.19; 1.61]), homelessness (4.87 [1.82; 13.02]), and reporting a feeling of loneliness (4.62 [3.06; 6.97]) were major predictors of suicide risk.

**Conclusions:**

Reducing the burden of precarious social conditions and discrimination is an important lever for preventing suicide risk among PLHIV in France. Comprehensive care models involving peer/community social interventions targeted at women and MSM need to be implemented to lower the risk of suicide in these specific subgroups of PLHIV.

## Introduction

In the early years of the HIV epidemic, suicide rates were very high among people living with HIV (PLHIV) due to poor prognosis [1]. These rates started to decrease with the arrival of effective and simplified antiretroviral regimens. Nevertheless, today in Western Europe, rates of suicide remain higher in PLHIV than in the general population [2]. Two European multi-cohort analyses documented relatively high rates of suicide in PLHIV: suicide accounted for 4% of all deaths in the D:A:D study in 2008 [3] and 6.4% of all deaths in the CASCADE study in 2006 [4]. Studies in both the pre- and post- highly-active antiretroviral treatment (HAART) eras have found that HIV disease progression and low CD4 cell count are associated with suicide [2,5]. Diagnosis of psychiatric illness is another significant correlate of suicide risk among PLHIV. In the Swiss HIV cohort study, a quarter of the individuals who died from suicide lived with an untreated psychiatric disorder (data from 1988 to 2008) [2]. In France, a study conducted in 2003 among PLHIV followed up in hospitals identified social vulnerability as a main correlate of attempted suicide in this population [6]. Given the global aging of PLHIV and the changes in the epidemiology of HIV infection observed in recent years [7], up-to-date knowledge of suicide risk factors among PLHIV is needed to better target individuals in most-at-risk subgroups. As the life expectancy of PLHIV increases to mirror that of the general population thanks to HAART, such information is essential to improve the management of HIV infection.

Using data from a national sample of PLHIV weighted to be representative of patients receiving care in French HIV outpatient services, this study aimed to identify the pattern of correlates of suicide risk currently observed among PLHIV in France.

## Material and methods

### The ANRS-VESPA2 French national survey

The ANRS-VESPA2 survey was conducted between April 2011 and January 2012 to describe the living conditions of a representative sample of adult PLHIV followed up in 73 French hospital departments delivering HIV care. A total of 3,022 individuals agreed to answer a face-to-face interview concerning their socio-demographic, behavioral and psychosocial characteristics. Hospital physicians providing care to the survey participants filled in a medical questionnaire which collected information about the patients' health status, history with HIV, comorbidities and prescribed treatments. Data were weighted and calibrated in order to be representative of all adult PLHIV followed up in French hospitals in 2011 and to take non-responders into account [8]. The ANRS-VESPA2 survey received approval from the French Advisory Committee on Information Processing in Material Research in the Field of Health (CCTIRS) and met the ethical requirements of the national French Data Protection Authority (CNIL). The survey was designed and performed in accordance with the Declaration of Helsinki, and all participants gave written informed consent.

### Study sample

The study sample included all participants in the ANRS-VESPA2 survey with available data concerning suicide thoughts, plans, and suicide attempts collected during the face-to-face interview, and with available medical data on comorbidities (N = 2,973).

### Variables

#### Outcome: Suicide risk

The face-to-face interview was used to assess suicide risk, which was defined as having either thought about and planned to commit suicide during the previous 12 months or attempted suicide during the same period of time [9]. In this study, suicide risk was thus conceptualized as suicide ideation and suicide attempt.

#### Explanatory variables

Socio-demographic characteristics tested for a possible relationship with suicide risk included gender and sexual orientation, age, nationality, living in a couple or not, educational level, employment status and housing occupancy status. Concerning gender and sexual orientation, the following three subgroups of PLHIV were distinguished: women, heterosexual men, and men who have sex with men (MSM). The latter category comprised all men self-identified as homosexual, bisexual or heterosexual who reported at least one male sexual partner during the previous 12 months [10]. Analyses were systematically adjusted for chronic infection with hepatitis C virus (HCV), which has been identified as a significant correlate of major depressive disorders in the French PLHIV population [11]. We assessed whether a history of injecting drug use (IDU) was associated with suicide risk, as injecting drug users are known to be at increased risk for mental health co-morbidities. Apart from chronic HCV infection, the other clinical characteristics examined to assess a possible relationship with suicide risk included chronic co-infection with hepatitis B virus (HBV), CD4 cell count at the most recent assessment, and a variable combining antiretroviral therapy (ART) regimen type and HIV plasma viral load. Tobacco use (with a cut-off at five cigarettes per day), alcohol consumption (assessed using the AUDIT questionnaire [12,13]) and drug use (opioids, stimulants, non-prescribed benzodiazepines and other drugs not including cannabis) were also examined. A binary variable for harmful alcohol consumption was defined with a cut-off of 16 for the AUDIT score [12]. Feelings of loneliness were assessed as a proxy of moral support received from PLHIV’s relatives and close social networks. Face-to-face interviews with PLHIV included the yes/no binary item "Do you feel lonely?" which was asked after another item which focused on individuals’ recent contact with family and friends and the extent to which these persons knew about their seropositivity. Finally, we examined the relationship between PLHIV’s experience of discrimination during the previous two years and suicide risk. Discrimination was documented in the following six social contexts: health services, work environment, job seeking, family, public services and spare time activities [14]. A global indicator of experience of discrimination (ranging from 0 to 6) was built, which accounted for the number of social contexts in which individuals reported discrimination.

### Statistical analyses

Poisson regression models were used to estimate the relationship between suicide risk and its potential correlates. Variables with a p-value <0.20 in the univariate analysis were considered eligible for multivariate testing. Multivariate analyses were systematically adjusted for chronic HCV co-infection. A stepwise backward selection procedure with a threshold at p = 0.05 was used to identify the other variables eligible to stay in the final multivariate model. All analyses were based on two-sided *p* values, with *p*<0.05 indicating statistical significance. They were conducted using Stata/SE 12.1 software for Windows (Stata Corp LP, USA).

## Results

### Description of the study sample

Overall 2,973 PLHIV out of 3,022 participants in the ANRS-VESPA2 survey (98%) were included in the study sample. Their main characteristics did not significantly differ from those of the other participants in the survey (data not shown).

**Fig 1:**
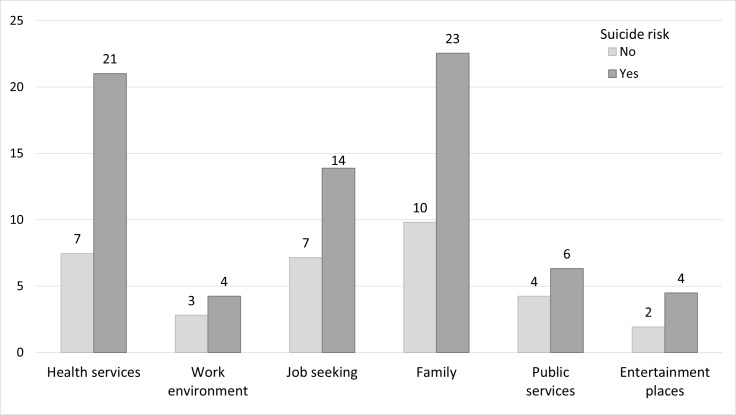
Experience of discrimination (in %) in six different social contexts during the previous two years as a function of suicide risk among people living with HIV followed up in French hospitals in 2011 (ANRS-VESPA2 national survey). Horizontal axis: social contexts of discrimination; vertical axis: Percentage of individuals (with or without suicide risk) who reported experience of discrimination in the specific social context during the previous two years.

Most individuals in the study sample were men (MSM: 39%, heterosexual men: 27%) and were over 40 years old (78%) (Table 1). Nine percent were chronically co-infected with HCV. More than one third reported feeling lonely, with a mean index of experience of discrimination of 0.36. Fig 1. presents the percentage of PLHIV who reported experiencing discrimination in each of the six different social contexts, as a function of suicide risk. The percentage of discriminated PLHIV–irrespective of the social context considered—was systematically higher among individuals with suicide risk than among the other PLHIV. Medical care (health services) and family were the two social contexts of discrimination most reported among PLHIV with suicide risk.

**Table 1:** Main characteristics of people living with HIV followed up in French hospitals in 2011 according to suicide risk (ANRS-VESPA2 national survey).

	All individuals (n = 2,973)	Suicide risk	
		No (93.7%)	Yes (6.3%)
	% of individuals or mean (SE)		
***Socio-demographic and economic characteristics***			
Gender and sexual orientation (ref. Heterosexual men)			
– MSM	39.3	93.3	6.7
– Women	33.3	92.1	7.9
Age–*years* (ref. > 60)	** **	** **	** **
– 18–29	4.7	94.4	5.6
– 30–39	17.5	94.8	5.2
– 40–49	36.2	91.7	8.3
– 50–59	28.2	94.1	5.9
Nationality (ref. French)	** **	** **	** **
– Non-French nationality from EU	2.7	97.2	2.8
– Non-French nationality not from EU	22.7	95.7	4.3
Not living in a couple	59.5	93.1	6.9
Educational level (ref. Higher than high school)	** **	** **	** **
– High school	10.8	95.6	4.4
– Lower than high school	58.6	93.0	7.0
Employment status (ref. Employed)	** **	** **	** **
– Economically inactive	36.0	93.0	7.0
– Unemployed	9.6	89.1	10.9
Housing occupancy status (ref. Owner/Tenant)	** **	** **	** **
– Free accommodation	10.5	90.4	9.6
– Tenant in residential care	0.6	79.5	20.5
– Homeless	0.6	77.5	22.5
***Clinical characteristics***			
Chronic HCV co-infection	9.0	89.6	10.4
Chronic HBV co-infection	4.3	97.0	3.0
CD4 cell count at the most recent assessment <200 *cells/mm*^*3*^	4.8	88.6	11.4
ART and HIV plasma VL (ref. Treated, undetectable VL)			
– Untreated	6.8	93.0	7.0
– Treated, no information on ART combination, detectable VL	1.3	87.3	12.7
– Monotherapy, detectable VL	0.3	91.4	8.6
– Bitherapy, detectable VL	1.0	93.9	6.1
– Multitherapy, detectable VL	16.9	91.7	8.3
***Addictive behaviors***			
History of IDU	10.4	89.7	10.3
Smoking > 5 cigarettes a day	25.9	90.6	9.4
Harmful alcohol consumption (AUDIT score ≥16)	2.5	86.2	13.8
Current drug consumption^1^	2.7	83.7	16.3
***Psychosocial characteristics***			
Feelings of loneliness	35.2	97.0	3.0
Experience of discrimination index^2^	0.36 (0.02)	0.33 (0.02)	0.72 (0.07)

^1^Among opioids, stimulants, non-prescribed benzodiazepines and other drugs not including cannabis

^2^Number of social contexts in which individuals experienced discrimination during the previous two years (varying between 0 and 6)

ART = antiretroviral therapy; AUDIT = alcohol use disorders identification test [12]; EU = European Union; HBV = hepatitis B virus; HCV = hepatitis C virus; IDU = injecting drug use; MSM = men who have sex with men; SA = sub-Saharan; SE = standard error; VL = viral load.

### Factors associated with suicide risk

Among the study sample, 6.3% of PLHIV reported suicide risk during the previous 12 months. In the univariate analyses, suicide risk was more frequent (p<0.20) in MSM and women, in individuals aged under 60 years old, those not living in a couple, those unemployed or inactive, French nationals and homeless people (Table 2). When considering clinical characteristics, patients with chronic HCV co-infection or those with a CD4 cell count < 200 cells/mm^3^ were significantly more likely to report suicide risk, while those with chronic HBV co-infection were less likely to do so. With respect to addictive behaviors, smoking at least five cigarettes per day, reporting harmful alcohol consumption and using drugs, were all associated with suicide risk. Finally, in terms of psychosocial characteristics, suicide risk was significantly higher in PLHIV who felt lonely and those who had experienced discrimination in a higher number of social contexts.

**Table 2:** Correlates of suicide risk in people living with HIV followed up in French hospitals in 2011 (ANRS-VESPA2 national survey).

	Univariate analysis	Multivariate analysis(n = 2,912)
	IRR [95% CI]	IRR [95% CI]
***Socio-demographic and economic characteristics***		
Gender and sexual orientation (ref. Heterosexual men)	*	*
– MSM	1.81 [1.14;2.90]	1.97 [1.22;3.19]
– Women	2.15 [1.33;3.47]	1.93 [1.17;3.19]
Age–*years* (ref. > 60)	*	
– 18–29	1.72 [0.75;3.96]	
– 30–39	1.62 [0.82;3.19]	
– 40–49	2.56 [1.41;4.67]	
– 50–59	1.83 [0.99;3.38]	
Nationality (ref. French)	*	*
– Non-French nationality from EU	0.40 [0.14;1.15]	0.24 [0.07;0.85]
– Non-French nationality not from EU	0.61 [0.40;0.93]	0.41 [0.26;0.65]
Not living in a couple	1.28 [0.89;1.85]◆	0.68 [0.47;0.98]*
Educational level (ref. Higher than high school)	◆** **	
– High school	0.78 [0.44;1.38]	
– Lower than high school	1.25 [0.86;1.79]	
Employment status (ref. Employed)	*	
– Economically inactive	1.41 [0.99;1.99]	
– Unemployed	2.20 [1.36;3.54]	
Housing occupancy status (ref. Owner/Tenant)	*	*
– Free accommodation	1.68 [1.12;2.53]	2.00 [1.36;2.93]
– Tenant in residential care	3.59 [1.28;10.12]	2.77 [0.92;8.34]
– Homeless	3.96 [1.52;10.29]	4.87 [1.82;13.02]
***Clinical characteristics***		
Chronic HCV co-infection	1.77 [1.06;2.95]*	1.70 [1.02;2.84]*
Chronic HBV co-infection	0.47 [0.17;1.28]◆	
CD4 cell count at the most recent assessment <200 *cells/mm*^*3*^	1.90 [1.02;3.52]*	1.72 [1.02;2.92]*
ART and HIV plasma VL (ref. Treated, undetectable VL)		
– Untreated	1.25 [0.63;2.47]	
– Treated, no information on ART combination, detectable VL	2.26 [0.96;5.34]	
– Monotherapy, detectable VL	1.53 [0.23;10.35]	
– Bitherapy, detectable VL	1.08 [0.36;3.22]	
– Multitherapy, detectable VL	1.49 [0.96;2.31]	
***Addictive behaviors***		
History of IDU	1.77 [1.08;2.88]*	
Smoking > 5 cigarettes a day	1.85 [1.34;2.57]*	
Harmful alcohol consumption (AUDIT score ≥16)	2.27 [1.11;4.65]*	
Current drug consumption^1^	2.72 [1.49;4.97]*	
***Psychosocial characteristics***		
Feelings of loneliness	4.94 [3.36;7.26]*	4.62 [3.06;6.97]*
Experience of discrimination index^2^	1.61 [1.40;1.85]*	1.39 [1.19;1.61]*

^1^Among opioids, stimulants, non-prescribed benzodiazepines and other drugs not including cannabis

^2^Number of social contexts in which individuals experienced discrimination during the previous two years (varying between 0 and 6)

ART = antiretroviral therapy; AUDIT = alcohol use disorders identification test [12]; CI = confidence interval; EU = European Union; HBV = hepatitis B virus; HCV = hepatitis C virus; IDU = injecting drug use; IRR = incidence rate ratio; MSM = men who have sex with men; SA = sub-Saharan; VL = viral load.

◆ p<0.20

* p<0.05 (Wald test)

Model fit statistic (link test: predicted values and squared predicted values coefficients and standard errors): Hat 0.30 (0.37) (*p = 0*.*42*); Hatsq -0.14 (0.07) (*p = 0*.*06*).

After adjustment for HIV immunological status and chronic HCV co-infection (Table 2), women (IRR [95%CI]:1.93 [1.17; 3.19]) and MSM (1.97 [1.22; 3.19]) presented a higher risk of suicide than the rest of the sample. Moreover, the number of social contexts in which discrimination was experienced (1.39[1.19; 1.61]), a feeling of loneliness (4.62 [3.06; 6.97]), and homelessness (4.87 [1.82; 13.02]) were major predictors of suicide risk.

## Discussion

This study provides an update of the main correlates of suicide risk in a representative sample of PLHIV followed up in French hospitals. Multivariate analyses identified social factors, including experience of discrimination and loneliness, as major correlates of suicide risk in this population, with MSM and women being the subgroups at higher risk of suicide.

Clinical variables significantly associated with suicide risk included low CD4 cell count and chronic HCV co-infection. A great body of research exploring the relationship between HIV status and suicide ideation/risk already exists [1,15–18]. The association between HCV infection and suicide risk has also been documented [6,19–22]. In the present study, impaired immunological status, defined as a CD4 cell count below 200 cells/mm^3^, was identified as a significant predictor of suicide risk. Results from the international literature differ concerning immunological status and suicide risk [23], with some authors finding that asymptomatic HIV-infected patients exhibit more suicidal behaviors than those with advanced HIV disease [24–26], while others find that increased CD4 cell count is associated with a lower risk of suicide [2,27].

The present study also identified MSM and women as two subpopulations of PLHIV at higher risk of suicide. This is consistent with previous literature which examined correlates of suicide attempts in the French PLHIV population [6]. Rates of HIV-positive women reporting suicide attempts as high as 26% have been reported in the international literature [28], with the risk of suicidal acts being greater in the period immediately following HIV diagnosis. Furthermore, there is epidemiological evidence that in general the risk of suicide attempts in MSM (particularly younger individuals) is higher, particularly in those living in social environments hostile to their sexual orientation [29]. In a case–control study based on the Australian HIV Observational Database [27], all cases of suicide or accidental/violent death in HIV-infected patients were in MSM/non-injecting or MSM/injecting drug users. In the present study, PLHIV with a history of IDU were at higher risk of suicide than others in the univariate analysis. However, a previous study conducted on the same study group using mediational analysis [30] showed that HCV infection, and not a history of IDU, is associated with higher risk of suicide. It is important to keep in mind that both IDU and MSM can suffer from substance-induced psychiatric disorders which often occur in association with alcohol consumption and other psychotropic drugs, and indeed with withdrawal from many substances, including stimulants. Recreational drug use and polydrug use are prevalent among HIV-positive MSM in Europe [31,32]. Today, addressing suicide risk in this population remains a major clinical concern in France as in many other countries, given that MSM continue to make up a very high percentage of new HIV cases [7].

Interestingly, the extent of discrimination was also a key correlate of suicide risk in our study. This is the first study to explore the association between suicide risk and experience of discrimination in different social contexts. It is worth noting that, even in a country like France, where HIV care is free and readily provided in specialized hospital outpatient services, discrimination by medical staff is reported by 8% of PLHIV receiving care [14]. Discrimination from family members is also frequent, despite the progressive “trivialization” of HIV infection observed in the French general population. In our study, health services and family were the two discrimination contexts most frequently reported by PLHIV with suicide risk (21% and 23% of individuals, respectively). Previous studies conducted among PLHIV showed that stigmatization within the family and in healthcare settings was more strongly related to psychological distress than stigmatization occurring in other social settings [33]. It is thus essential to develop interventions targeted at these influential settings to relieve the burden of stigma and discrimination among PLHIV. Patient-perceived experience of rejection behaviors by medical staff has also been shown to be associated with impaired physical and mental health-related quality of life among French PLHIV [34]. Taken together, these results underline the need for ongoing training of health staff which encourages the adoption of an empathic approach to care for HIV-positive patients. Improving health care providers’ communication skills, and fostering openness about sexual and reproductive health topics in HIV care may also help to relieve the burden of stigma and discrimination for PLHIV [35]. In addition, it is essential to involve family members in HIV care and support to PLHIV through adequate educational interventions.

Social isolation, expressed by feelings of loneliness, was a significant predictor of suicide risk, even after adjustment for homelessness which is a major correlate of suicide risk. By contrast, individuals not living in a couple were less concerned by suicide risk. Paradoxical as it may seem, this result indirectly suggests that PLHIV may face difficulties when coping with their HIV status in the context of intimate relationships. Psychosocial support, counseling and contact with PLHIV associations can help to put an end to feelings of loneliness.

The strong and independent associations found between homelessness or lack of social support and suicide risk highlight the importance of a series of psychosocial interventions delivered by some Non-Governmental Organizations (NGOs). Such NGOs offer “therapeutic apartments” which include lodging, psychosocial interventions and medical care for individuals accumulating multiple vulnerabilities (ex: homelessness, HIV, psychiatric disorders etc). Improved scaling-up of these interventions has the potential to reduce suicide risk in PLHIV living in poverty and experiencing isolation.

In line with previous studies [36–41], we also found that tobacco smoking and harmful alcohol consumption were associated with suicide risk in the univariate analysis. However, these associations were no longer significant in the multivariate analysis. This may be explained by the association between tobacco smoking and HCV co-infection [42] on the one hand, and between alcohol use and psychosocial characteristics including feelings of loneliness and discrimination on the other.

Among the limitations of the study, we presume that the prevalence of suicide risk was underestimated by social desirability bias due to self-reporting. In addition, our study sample only included people receiving HIV care in French hospital departments and therefore cannot be deemed to be representative of all PLHIV living in France. However, in France, the great majority of PLHIV is followed-up in specialized hospital services, which suggests that our results can be extrapolated to most PLHIV living in the country [43].

## Conclusions

Women and MSM present a non-negligible risk of suicide among PLHIV in France. Targeted comprehensive care models involving peer/community social interventions need to be implemented immediately in these populations. Reducing the burden of precarious social conditions and discrimination may be an important lever for preventing suicide risk among PLHIV.

## Supporting information

S1 Table Characteristics of people who reported suicide attempt during the previous 12 months (ANRS-VESPA2 national survey). ^1^Among opioids, stimulants, non-prescribed benzodiazepines and other drugs not including cannabis.
^2^Number of areas in which individuals experienced discrimination during the previous two years (varying between 0 and 6).
ART = antiretroviral therapy; AUDIT = alcohol use disorders identification test [12]; CI = confidence interval; EU = European Union; HBV = hepatitis B virus; HCV = hepatitis C virus; IDU = injecting drug use; MSM = men who have sex with men; SA = sub-Saharan; SE = standard error; VL = viral load.
(DOCX)
