# Supplementary material for: Suicide risk in a representative sample of people receiving HIV care: Time to target most-at-risk populations (ANRS VESPA2 French national survey)
Source: PLoS One. 2017 Feb 13;12(2):e0171645. doi: 10.1371/journal.pone.0171645 (PMC5305195; doi:10.1371/journal.pone.0171645)
Supplement: S1 Table — 1Among opioids, stimulants, non-prescribed benzodiazepines and other drugs not including cannabis. 2Number of areas in which individuals experienced discrimination during the previous two years (varying between 0 and 6). ART = antiretroviral therapy; AUDIT = alcohol use disorders identification test [12]; CI = confidence interval; EU = European Union; HBV = hepatitis B virus; HCV = hepatitis C virus; IDU = injecting drug use; MSM = men who have sex with men; SA = sub-Saharan; SE = standard error; VL = viral load. (DOCX) [file pone.0171645.s001.docx]

**S1. Characteristics of people who reported suicide attempt during the previous 12 months (ANRS-VESPA2 national survey)**

|  | **Suicide attempt during the previous 12 months** | | *p-value*  *(Wald test)* |
| --- | --- | --- | --- |
|  | No (98.7%) | Yes (1.3%) |  |
|  | Column % of individuals or mean (SE) | | |
| ***Socio-demographic and economic characteristics*** |  |  |  |
| Gender/transmission category (ref. Heterosexual men) |  |  | *0.01* |
| MSM | 39.2 | 46.4 |  |
| Women | 33.1 | 46.0 |  |
| History of IDU | 10.3 | 12.5 | *0.71* |
| Age – *years* (ref. > 60) |  |  | *0.01* |
| 18-29 | 4.7 | 3.3 |  |
| 30-39 | 17.3 | 33.9 |  |
| 40-49 | 36.0 | 49.9 |  |
| 50-59 | 28.5 | 11.5 |  |
| Nationality (ref. French) |  |  | *0.47* |
| Non-French nationality from EU | 2.7 | 1.1 |  |
| Non-French nationality not from EU | 22.7 | 20.9 |  |
| Not living in a couple | 59.4 | 72.1 | *0.16* |
| Educational level (ref. Higher than high school) |  |  | *0.11* |
| High school | 10.9 | 9.3 |  |
| Lower than high school | 58.4 | 74.8 |  |
| Employment status (ref. Employed) |  |  | *0.25* |
| Inactive | 36.1 | 25.6 |  |
| Unemployed | 9.5 | 18.5 |  |
| Housing occupancy status (ref. Owner/Tenant) |  |  | *0.52* |
| Free accommodation | 10.5 | 7.0 |  |
| Tenant in residential care | 0.6 | 2.8 |  |
| Homeless | 0.6 | 2.0 |  |
| ***Clinical characteristics*** |  |  |  |
| Chronic HCV co-infection | 8.9 | 18.1 | *0.42* |
| Chronic HBV co-infection | 4.3 | 4.7 | *0.90* |
| CD4 cell count at the most recent assessment <200 *cells/mm^3^* | 4.8 | 3.5 | *0.56* |
| ART and HIV plasma VL (ref. Treated, undetectable VL) |  |  | *0.03* |
| Not treated | 6.7 | 14.7 |  |
| Treated, no information on ART combination, detectable VL | 1.4 | 0.0 |  |
| Monotherapy, detectable VL | 0.3 | 2.0 |  |
| Bitherapy, detectable VL | 1.0 | 2.0 |  |
| Multitherapy, detectable VL | 16.9 | 18.8 |  |
| ***Addictive behaviors*** |  |  |  |
| Smoking > 5 cigarettes a day | 25.8 | 36.3 | *0.18* |
| Harmful alcohol consumption (AUDIT score ≥16) | 2.4 | 6.7 | *0.40* |
| Current drug consumption^1^ | 2.7 | 2.2 | *0.79* |
| ***Psychosocial characteristics*** |  |  |  |
| Feelings of loneliness | 34.5 | 81.0 | *<0.001* |
| Experience of discrimination index^2^ | 0.35 (0.02) | 0.94 (0.24) | *0.02* |
